# Estimating Radicle Length of Germinating Elm Seeds via Deep Learning

**DOI:** 10.3390/s25165024

**Published:** 2025-08-13

**Authors:** Dantong Li, Yang Luo, Hua Xue, Guodong Sun

**Affiliations:** 1 School of Information and AI, Beijing Forestry University, Beijing 100083, China; audrey606@bjfu.edu.cn (D.L.); luoyang@bjfu.edu.cn (Y.L.); 2 Hebei Key Lab of Smart National Park, Beijing Forestry University, Beijing 100083, China; 3 School of Biological Sciences and Technology, Beijing Forestry University, Beijing 100083, China; xuehua2013@bjfu.edu.cn

**Keywords:** elm seeds, seed vigor, radicle length, deep learning

## Abstract

Accurate measurement of seedling traits is essential for plant phenotyping, particularly in understanding growth dynamics and stress responses. Elm trees (*Ulmus* spp.), ecologically and economically significant, pose unique challenges due to their curved seedling morphology. Traditional manual measurement methods are time-consuming, prone to human error, and often lack consistency. Moreover, automated approaches remain limited and often fail to accurately process seedlings with nonlinear or curved morphologies. In this study, we introduce GLEN, a deep learning-based model for detecting germinating elm seeds and accurately estimating their lengths of germinating structures. It leverages a dual-path architecture that combines pixel-level spatial features with instance-level semantic information, enabling robust measurement of curved radicles. To support training, we construct GermElmData, a curated dataset of annotated elm seedling images, and introduce a novel synthetic data generation pipeline that produces high-fidelity, morphologically diverse germination images. This reduces the dependence on extensive manual annotations and improves model generalization. Experimental results demonstrate that GLEN achieves an estimation error on the order of millimeters, outperforming existing models. Beyond quantifying germinating elm seeds, the architectural design and data augmentation strategies in GLEN offer a scalable framework for morphological quantification in both plant phenotyping and broader biomedical imaging domains.

## 1. Introduction

Seed vigor is a fundamental indicator of seed quality, reflecting a seed’s ability to germinate rapidly and uniformly and to develop into healthy seedlings under diverse environmental conditions [1]. Germination performance is a primary manifestation of seed vigor and is typically assessed using parameters such as germination rate, germination potential, and the vigor index [2,3]. Among these, the vigor index is widely used as a composite metric, typically calculated as the product of germination potential and the combined length of radicle and hypocotyl. Therefore, accurate measurement of these elongating germination structures is essential for a reliable assessment of seed vigor. Beyond its role in vigor evaluation, radicle and hypocotyl length also serve as a key phenotypic trait in developmental and physiological studies, with broad applications in agriculture, including crop improvement, ecological monitoring, and the modeling of plant responses to environmental stress and climate change [4,5,6,7,8].

Among dicotyledonous plants, the elm tree (*Ulmus* spp.) is an ecologically and economically important tree widely distributed across temperate regions [9,10]. Elm trees contribute substantially to ecosystem services by stabilizing soil structures, offering habitat diversity, and supporting urban sustainability through shade provision and pollution mitigation [11]. Economically, elm timber holds considerable value, frequently utilized in furniture production, flooring, veneer, and other wood products [12,13]. Furthermore, elm species serve as bioindicators of environmental health, notably reflecting urban air quality [14]. Given these multifaceted roles, reliably measuring germination and early elm seedling growth traits is essential for breeding disease-resistant cultivars, directing conservation efforts, and promoting sustainable forestry practices.

Conventionally, seedling germination parameters, including radicle and hypocotyl lengths, are predominantly quantified using traditional manual and semi-automated methods, with manual approaches still widely employed. For instance, studies have employed digital calipers [15], vernier calipers [16,17], and calibrated rulers [7], alongside other manual techniques documented in multiple studies [8,18,19,20]. While straightforward, these manual methods are labor-intensive, prone to human error due to inherent operator dependency, and inherently inefficient, particularly at large scales.

To address these shortcomings, recent years have seen growing adoption of semi-automated, image-based phenotyping approaches [21,22,23,24,25,26,27]. These methods leverage image-processing software to extract length data from digitized seedlings, typically obtained via infrared imaging, flatbed scanning, or high-resolution photography (e.g., using an EPSON flatbed scanner [27]). Operators then manually trace structures like the radicle or hypocotyl using tools like ImageJ  [6,20,27,28], and the software converts pixel distances to physical length. General experimental setups for such image-based measurements have also been described [29].

Although reducing labor and improving consistency compared to purely manual methods, these semi-automated approaches still require manual intervention (specifically the tracing step) and thus cannot be considered fully automated. Furthermore, they inherently assume seeds/seedlings lie flat and can be manually traced without loss of spatial detail. Consequently, they are poorly suited for measuring curved or structurally complex seedlings, such as the curved radicles commonly observed in elm seedlings. Their accuracy is significantly undermined by natural morphological variability (e.g., pronounced curvature) and challenging imaging conditions (e.g., inconsistent illumination, scale variations), which impede accurate pixel-to-length conversion without manual oversight.

Recent advances in deep learning have significantly propelled precision agriculture and plant phenotyping [30]. Much of the literature focuses on employing models like convolutional neural networks (CNNs) [31,32,33,34], Transformers [35], or their variants [36] for seed species classification tasks, achieving high accuracy. However, length estimation of germinating structures presents distinct challenges beyond classification, specifically the need to account for natural curvature, variations in image scale, and the accurate conversion of pixel measurements to real-world units. Prior research has given limited attention to these complexities for germination traits.

Only recently have studies applied deep learning models such as U-Net [37], improved U-Net [38], DeepLabV3+ [39], or CNN [40] primarily for segmentation tasks targeting hypocotyls, rice seedlings, or pine (*P. radiata*) embryos. In these cases, the typical workflow involves first segmenting the seedling image to identify the structure of interest, followed by applying separate algorithms (like pixel counting) to convert the segmented regions into quantitative length measurements. This pipeline constitutes a segmentation-plus-conversion approach, not a fully end-to-end solution. Crucially, these methods rely on simplified two-dimensional assumptions and are ill-suited for capturing the three-dimensionally curved morphologies characteristic of dicotyledonous seedlings like elm. Consequently, they often yield substantial errors in both segmentation and subsequent length estimation under real-world conditions.

This underscores the urgent need for robust, fully automated, end-to-end methodologies capable of accurately quantifying germination lengths directly from images across diverse morphologies (including significant curvature) and variable imaging conditions, without requiring manual intervention or intermediary conversion steps.

To address these limitations, we propose GLEN (*g*ermination *len*gth measurement), a deep learning-based model specially designed to automate detection and germination length estimation of elm seedlings. As an end-to-end model, GLEN integrates multi-level visual and semantic feature extraction to accurately capture seedling region and morphological complexities—challenges inadequately addressed by existing pixel-level classification methods. This paper presents threefold contributions. First, we construct GermElmData, a dataset comprising high-resolution images of germinating elm seeds annotated with morphological details. Recognizing that accurate length estimation relies on precise segmentation of seedling instance, we address the data-dependency challenge by introducing a synthetic data generation pipeline powered by artificial intelligence (AI), which produces high-fidelity synthetic images closely resembling natural elm seedling morphologies. This approach not only enhances GLEN’s segmentation capabilities but also offers a scalable and efficient strategy for morphological data augmentation, broadly applicable to other phenotyping tasks. Second, we propose a novel deep learning architecture, GLEN, that advances beyond conventional pixel-level classification of seed species. GLEN employs a dual-path design that orchestrates dense spatial feature extraction and instance-level semantic learning, enabling direct regression from pixel data to length measurements—without human intervention or the need for additional algorithms. This architecture adopts the joint modeling of fine-grained structural details and global curvature trends, yielding robust performance across various seedling shapes and orientations. More broadly, the architectural principle of GLEN could offer a generalizable framework that can be extended to other plant phenotyping and bio-image analysis requiring precise morphological quantification. Third, extensive evaluation on the GermElmData shows that GLEN achieves a mean absolute error on the order of millimeters in measuring curved elm radicles, significantly outperforming conventional approaches such as U-Net and setting a new benchmark for seedling morphological monitoring.

The remainder of this paper is organized as follows. Section 2 details the development of a curated image dataset of elm seedlings. Section 3 details the design and components of the proposed GLEN architecture. Section 4 presents experimental evaluation. Section 5 discusses the GLEN’s strengths, limitations, and practical insights. Section 6 concludes this work and highlights future directions.

## 2. Dataset Creation

We used elm tree seeds (Beijing Forestry University Campus, Beijing, China) as experimental material. These seeds exhibit morphological and developmental characteristics of dicotyledonous plants, making them a representative model for studying traits common to many dicotyledonous seedlings. Seeds were collected from their natural habitat in May 2024 and stored at ${-4\;}^{\circ }$C to preserve viability and germination potential. Prior to experimentation, seeds were removed from storage and placed in sterilized glass petri dishes (18 mm in height, 44.5 mm in inner radius) containing an appropriate volume of purified water to facilitate germination at room temperature.

**Figure 1:**
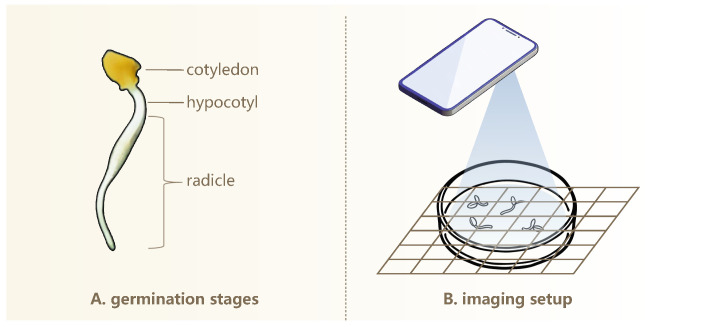
Morphological appearance of elm seed’s germinating structure (**A**) and the imaging setup (**B**).

As illustrated in Figure 1A, during elm seed germination, the seed extends both the hypocotyl and radicle. The radicle rapidly elongates and becomes the most prominent structure, whereas the hypocotyl grows more slowly and typically remains short, often rendering it reasonably negligible in length. Radicle elongation is highly dynamic and sensitive to external environmental conditions, and thus, its length serves as a meaningful indicator of seedling vigor and developmental potential [41,42]. For simplicity, we use “radicle” to refer to the combined radicle and hypocotyl structure.

### 2.1. Image Acquisition

We captured high-resolution images of germinating seeds using the camera of a realme GT2 Master Exploration Edition smartphone (manufacturer: realme, Shenzhen, China) equipped with a SONY IMX766 sensor (Sony Corporation, Tokyo, Japan; 50 megapixels). This setup enables detailed visualization of morphological features during seed germination. Prior to each imaging session, germinating seeds were transferred to fresh, sterilized petri dishes to prevent condensation interference and ensure high-quality, unobstructed images of seedling growth patterns. Figure 1B illustrates the imaging setup used to capture top-down photographs of germinating seeds arranged in petri dishes under ambient room lighting. The camera was positioned around 20 cm above the dishes. Minor variations in the phone’s height are acceptable, provided that the entire dish is clearly visible in the image. This simplifies the image acquisition process and increases overall efficiency. To enhance visual contrast and facilitate subsequent analysis, three distinct background colors were used: black, white, and natural wood. Additionally, two imaging conditions were included—with and without grid paper printed with a 1cm×1cm scale—to accommodate different analytical requirements.

### 2.2. Annotation

In all GermElmData images, each germinating seed’s radicle structure is annotated with three types of information labels: an instance segmentation mask, ground-truth length measurement (in real-world units), and corresponding pixel-based length values.

**Figure 2:**
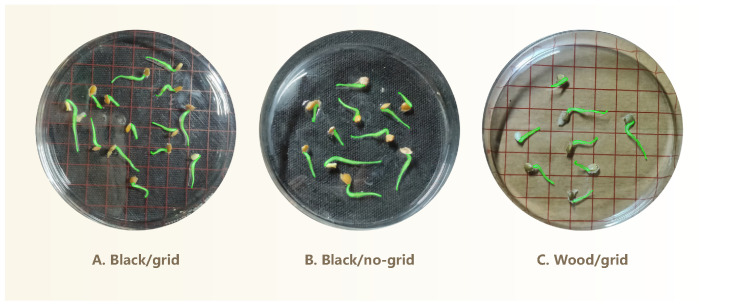
Example of instance mask annotations (green regions) for elm seedling’s radicle.

**Instance mask**. In visual semantic segmentation, an instance mask is a pixel-level representation that not only classifies object regions, but also distinguishes individual instances of the same class. As shown in Figure 2, all elm radicles are annotated with an instance mask. In this study, semantic segmentation was performed using ISAT, a semi-automated annotation tool [43] that integrates Meta’s Segment Anything Model (SAM) [44] into the polygon-based annotation workflow of LabelMe [45]. This hybrid approach facilitates morphologically accurate and efficient annotations. These seedling masks enable us to reliably identify and extract the radicle regions from images, enabling downstream length estimation.

**Ground-truth length**. We manually annotated each germinating seed with its radicle length to provide ground-truth measurements for model training. Due to the naturally curved morphology of radicles, traditional ruler-based measurements are unsuitable. To measure radicle length accurately, we employed a non-elastic cotton string (approximately 1 mm in diameter), carefully aligning it closely along the full structure of each radicle. The length of the straightened string was then measured using a ruler. Each measurement was repeated three times, and the average value was recorded as the final ground-truth annotation for radicle length.

**Pixel-based length**. Variations in camera distance during image capture can cause the same seedling to appear larger or smaller in pixel dimensions. This discrepancy introduces inconsistency in absolute length measurements across images. Feeding these inconsistent values into a learning model may lead to incorrect associations between visual features and true morphological traits. To address this issue, we adopted a pixel-based approach, allowing GLEN to learn relative morphological characteristics consistently across varying image scales. This normalization ensures that the interpretation of seedling features remains accurate and robust, regardless of differences in capture distance. Let $\ell_{h}^{\mathrm{GT}}$ denote the ground-truth radicle length of a seedling instance, and let $\ell_{g}^{\mathrm{PIX}}$ denote the pixel edge length of the background grid. Given a seedling image, the corresponding pixel-based length of $\ell_{h}^{\mathrm{GT}}$ can be derived by

(1) $$\ell_{h}^{\mathrm{PIX}}=\frac{\ell_{h}^{\mathrm{GT}}\cdot \ell_{g}^{\mathrm{PIX}}}{\Delta s_{g}}\;,$$

where $\Delta s_{g}$ is the physical side length (i.e., 10 mm) of the background grid. To calculate $\ell_{g}^{\mathrm{PIX}}$, we randomly selected five grids from each image and measured their side lengths using a custom-built Python-based interactive tool. For each selected side, this tool recorded the coordinates of its endpoints through mouse clicks and calculated the Euclidean distance using NumPy’s linear algebra module (i.e., numpy.linalg.norm [46]). To mitigate potential errors arising from manual annotation, the final $\ell_{g}^{\mathrm{PIX}}$ value for each image was calculated as the average of five independent measurements.

### 2.3. Descriptive Statistics

**Figure 3:**
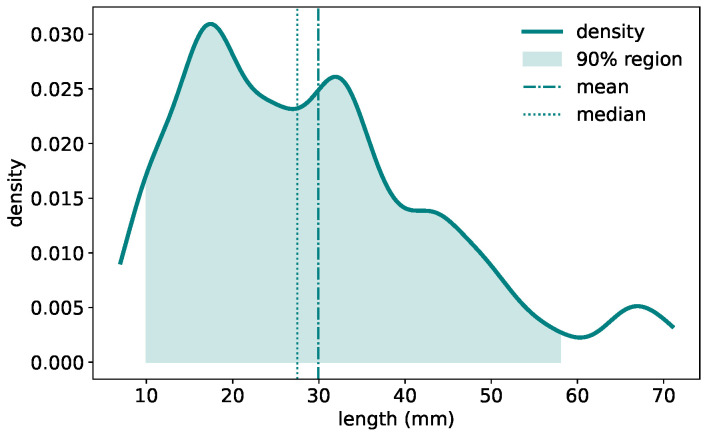
Distribution of radicle length values of elm seeds in GermElmData.

The GermElmData dataset comprises 796 images of germinating elm seeds and features morphological diversity associated with elm seed germination. Figure 3 presents the distribution of radicle lengths. The density plot reveals a right-skewed distribution. The majority of samples fall within the 10∼50 mm range, with a peak frequency around 18∼20 mm. The mean radicle length is close to 30 mm, while the median is slightly lower at 28 mm. This variation in radicle length exhibits the morphological diversity captured in the dataset and may reflect underlying genetic or environmental influences on seedling development.

**Figure 4:**
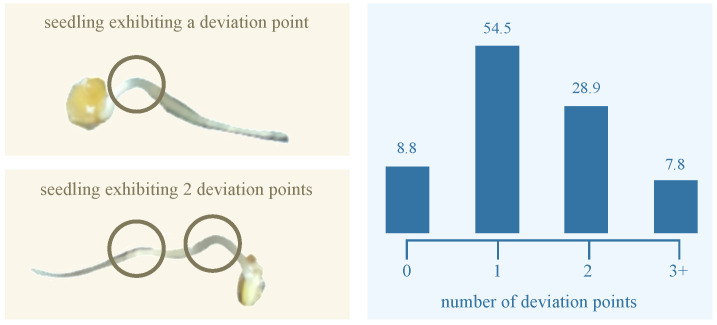
Illustration of deviation points and their distribution in GermElmData.

Typically, the radicle structure of the germinating seed does not remain or grow entirely flat on the dish surface; instead, it often exhibits three-dimensional curvature during development. To quantify this curvature, we identified *deviation points*—local maxima where the radicle visibly lifts from dish surface. Based on the number of such deviation points, we classified each radicle into one of four curvature categories: (1) no deviation, (2) one deviation point, (3) two deviation points, and (4) more than two deviation points. Figure 4 illustrates the distribution of these curvature categories among germinating elm seeds. The majority (54.5%) fall into the one-deviation-point category. Fewer than 9% of radicles grew flat and exhibited no deviation. The smallest group (7.8%) comprised radicles with more than two deviation points, reflecting more complex growth structures. This distribution shows the morphological variability in elm seed radicle development and underscores the importance of curvature-aware phenotyping in seedling analysis. Overall, our GermElmData dataset effectively captures diverse morphological traits, providing robust training and validation data for subsequent GLEN training.

To quantitatively validate the observed correlation between radicle length and curvature complexity revealed in our dataset analysis, we employed non-parametric correlation methods specifically chosen for their suitability to discrete ordinal data. Using Spearman’s rank-order correlation, which assesses monotonic relationships through rank differences, and Kendall’s Tau, which evaluates concordance probabilities between paired observations, our analysis demonstrated a statistically significant strong positive association, with Spearman’s ρ=0.839 (*p* < 0.0001) and Kendall’s τ=0.741 (*p* = 0.0002). This confirms that longer radicles exhibit significantly more complex curvature patterns characterized by increased deviation points.

### 2.4. Synthetic Data Supporting Radicle Detection

From a computer vision perspective, accurate measurement of radicle length by a learning model depends critically on the accurate detection of the radicle region. In practice, however, this task is complicated by the curved morphology of elm seedlings and subtle color variations that reduce contrast between adjacent parts of seedling. These difficulties are further exacerbated under limited training data conditions. To address this issue, we developed an AI-driven synthetic dataset specifically aimed to improve the detection accuracy of radicle regions in data-scarce scenarios. The synthetic dataset was generated through the following procedure.

**Morphological guidance**. We generated synthetic radicle images using Dreamina, which is an AI image generation tool developed by ByteDancing [47]. After uploading a source image to Dreamian, users can input text prompts or provide arrow-based guidance for localized refinement, resulting in the generation of synthetic target images.

**Figure 5:**
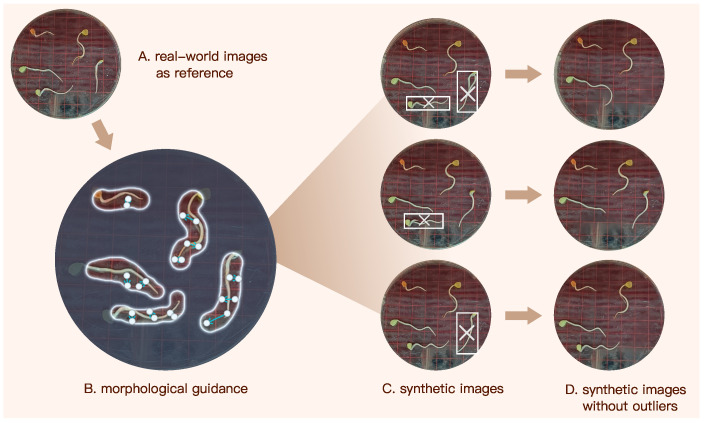
AI-driven procedure of synthesizing images of elm seedlings.

Figure 5 illustrates the pipeline of generating our synthetic image dataset using Dreamina. We first input Dreamina a real-world image capturing elm seedlings, and then manually circled each radicle. Subsequently, we annotated each radicle with arrows that indicate its morphological orientation and curvature, as illustrated in Figure 5B. These annotations provide Dreamina with explicit shape and curvature guidance, enabling it to regulate axial elongation trajectories and curvature patterns. As a result, the generated synthetic radicles closely approximate the morphology of their real counterparts.

**Outlier removal**. While Dreamina can rapidly synthesize images based on user prompts, a small subset of synthetic radicles deviated noticeably from realistic morphology. As shown in Figure 5C, for instance, a few number of synthetic seedlings exhibit abnormal features such as multiple radicles or unnatural transitions between the cotyledon and radicles regions. These are treated as outliers. To address this, each synthetic image was manually inspected, and outlier instances were removed using Dreamina’s eraser or any other AI-erasers that can eliminate undesired content while preserving the original background pattern.

After outlier removal, the remaining synthetic seedlings closely resemble their real counterparts, making it difficult to distinguish them at first glance. Although Dreamina effectively synthesizes germinating elm seed images, it does not embed explicit length information in the generated results, as no such prompts were provided. However, this limitation does not compromise the performance of our model in length prediction, as the synthetic dataset is used solely to enhance radicle detection accuracy. The final synthetic dataset comprises 655 seedlings with instance masks, expanding the size and diversity of our training data for radicle detection.

### 2.5. Generalization Analysis of Synthetic Data

To evaluate the domain gap between synthetic and real images, we conducted a comparative experiment. Using 500 instances each of real images and synthetic images, we trained identical GLEN models (all hyperparameters held constant) and evaluated segmentation performance on the same real-image validation set. As shown in Table 1, the real-image-trained model achieved 81.1% bbox AP50 and 79.4% segm AP50, while the synthetic-image-trained model attained 77.4% and 74.9%, respectively. Although synthetic data yields marginally lower performance, its segmentation accuracy exceeds 74%—demonstrating effective learning of radicle morphological features. This performance difference primarily stems from distribution shifts in texture details and illumination conditions between synthetic and real images. In this study, synthetic data complements real data to improve model generalization capability.

## 3. Model Proposed

In this section, we first introduce the fundamental concept underlying the GLEN model, then describe its architectural design in detail, and finally outline the training procedure for optimizing model performance.

### 3.1. Overall Architecture of GLEN

**Figure 6:**
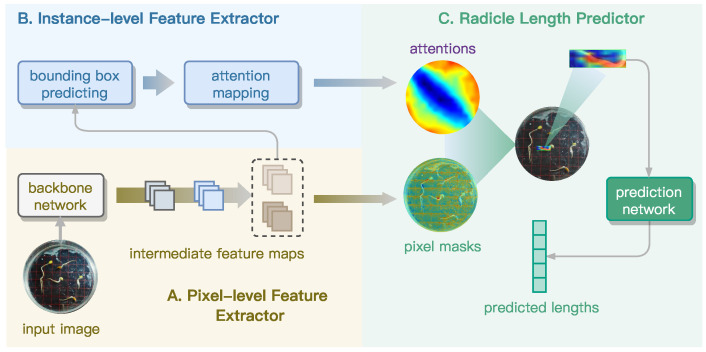
Overall architecture of GLEN including three primary modules.

The proposed GLEN model is a novel and end-to-end deep learning framework designed for fine-grained prediction of radicle length through a structured, multi-stage feature extraction process. Unlike conventional computer vision-based models for germination length estimation, which primarily rely on pixel-level feature extraction and classification, GLEN introduces a unified dual-path architecture, inspired by the BlendMask model [48], and then synergistically integrates pixel-level granularity with high-level instance abstraction. This design allows GLEN to effectively handle the complex morphology of curved radicles. As illustrated in Figure 6, GLEN comprises three coordinated modules:

- A *Pixel-level Feature Extractor* that constructs a feature pyramid from a ResNet-50 backbone through three sequential stages, capturing both low- and high-level features at the pixel level. This module is further enhanced with Squeeze-and-Excitation blocks, which recalibrate channel-wise feature responses guided by low-level feature masks derived from the input images.
- An *Instance-level Feature Extractor* that localizes individual object instances (i.e., seed radicle) by predicting bounding boxes and generating instance-specific attention masks for all radicles in the input image.
- A *Radicle Length Predictor* that mixes the pixel-level and instance-level features, leveraging the mixed representation together with a feature mask selected from the low-level feature pyramid to rapidly and accurately perform radicle length regression.

### 3.2. Pixel-Level Feature Extraction

**Figure 7:**
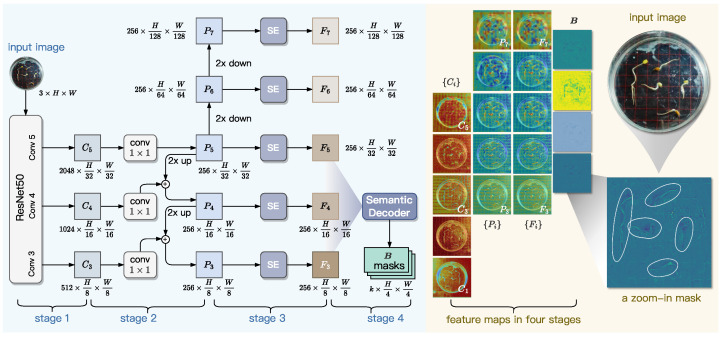
Structure of the Pixel-level Feature Extractor in GLEN (**left**) and representative feature maps extracted from four sequential stages (**right**), where feature maps at each level are aggregated across channels to generate a single merged map for convenient visualization.

As shown in Figure 7, the Pixel-level Feature Extraction module employs a ResNet+FPN backbone to extract multi-scale features from input images. The ResNet component generates intermediate feature maps, which are then transformed by a feature pyramid network (FPN) into a multi-level representation with uniform channel dimensions. To enhance feature quality, Squeeze-and-Excitation (SE) blocks are applied for channel-wise recalibration. A pixel-level decoder subsequently aggregates the multi-scale features to produce instance-agnostic bases that capture both universal semantic patterns and position-sensitive spatial details. These bases serve as foundational components for generating instance masks through attention-guided blending mechanisms. This design efficiently fuses high-resolution localization cues from lower-level features with rich contextual semantics from higher levels, while maintaining computational efficiency.

#### 3.2.1. Backbone Network

In computer vision tasks, ResNet and FPN are two widely adopted approaches to deep feature extraction and multi-scale feature aggregation, respectively. Specifically, ResNet extracts hierarchical features through residual blocks from bottom to top, while FPN refines and aggregates these features through lateral connections from top to bottom. Their collaboration can enhance multi-scale feature representation by combining high-resolution shallow features with semantically rich deep features [49,50].

In GLEN, ResNet-50 consists of five hierarchical steps that progressively extract features from an input image $I\in R^{H\times W\times c}$, where *H* and *W* denote the height and width of image *I* in pixels, and *c* denotes the number of channels of *I*. At each step *i* for 1≤i≤5, ResNet-50 outputs a feature map, denoted as $C_{i}$. Each subsequent feature map exhibits a two-fold reduction in spatial resolution relative to its predecessor, enabling the ResNet-50 network to capture increasingly abstract representations across scales. We found that $C_{1}$ and $C_{2}$ are high in spatial density, which incurs substantial computational costs for subsequent processing, while contributing less texture and boundary information. Therefore, in the design of GLEN, we only select three feature maps, $\left\{C_{3},C_{4},C_{5}\right\}$, which collectively maintain sufficient spatial resolution for downstream generation of top-down feature maps.

To further improve feature hierarchy integration across scales, we armor ResNet50 with a FPN component. As shown in Stage 2 of Figure 7, FPN constructs a pyramid-like set of multi-scale feature maps, denoted as $\left\{P_{i}\right\}$, based on the feature maps $\left\{C_{3},C_{4},C_{5}\right\}$. This construction process begins at the highest-level feature map $C_{5}$. Specifically, a 1×1 convolution is applied to $C_{5}$ to produce the feature map $P_{5}$, preserving the spatial resolution and regularizing the channel dimension to 256. Subsequently, $P_{6}$ is constructed by downsampling $P_{5}$ with a factor of two, and $P_{7}$ is similarly obtained by downsampling $P_{6}$. To generate $P_{4}$, the next-level feature map below $P_{5}$, we upsample $P_{5}$ with a factor of two and perform element-wise addition between the upsampled $P_{5}$ and ${\mathrm{conv}}_{1\times 1}\left(C_{4}\right)$. Following the same procedure, we sequentially generate $P_{3}$. This process yields the final set of feature maps $\left\{P_{3}\cdots P_{7}\right\}$, collectively forming a pyramid-shaped multi-scale representation. For 3≤i≤6, each feature map $P_{i}$ at the *i*-th pyramid level is twice the spatial resolution of its predecessor $P_{i+1}$.

#### 3.2.2. SE Block

The standard FPN applies identical weights to all feature channels during fusion, lacking the capacity to selectively enhance features from convolutional kernels that capture different spatial details. This uniform blending causes progressive loss of critical edge information, particularly eroding edge features critical for detecting low-contrast biological structures like seed radicles. The inherent channel redundancy induces a weakening of morphological signals and amplification of noise during the upsampling process. These limitations result in broken edge predictions and reduced robustness in challenging backgrounds, which significantly decreases accuracy in tasks that require precise detection of slender structures.

To enhance the representational capacity of multi-scale features and address channel redundancy in conventional FPN, lightweight Squeeze-and-Excitation (SE) blocks are incorporated at each pyramid level, as shown in Stage 3 of Figure 7. This mechanism automatically learns channel-wise importance coefficients by analyzing inter-channel correlations, thereby adaptively enhancing discriminative features while suppressing non-essential ones. For an input feature map *P* from any pyramid layer, the SE block performs channel-wise feature recalibration through three sequential operations: *squeeze*, *excitation*, and *scale*. The squeeze operation aggregates the global spatial information of *P* by performing average pooling on each channel; this calculation across all channels is written as

(2) $$z=\frac{1}{H\times W}\sum_{i=1}^{H}\sum_{j=1}^{W}P\left(i,j\right)\;,$$

where P(i,j) denotes the pixel value located at the *i*-th row and *j*-th column of the feature map *P*, while *H* and *W* are the height and width of *P*, respectively. The squeeze outputs a *c*-dimensional vector z, where *c* is the number of channels of *P*. Then, the excitation operation performs adaptive channel-wise weighting via a gated mechanism, calculated by

(3) $$s=\mathrm{sigmoid}(W_{2}\cdot \mathrm{ReLU}\left(W_{1}\cdot z\right))\;,$$

where $W_{1}$ and $W_{2}$ are the learnable parameters for the SE block. Here, $W_{1}$ is a continuous matrix of size $\frac{c}{r}\times c$, while $W_{2}$, of size $c\times \frac{c}{r}$, where *r* is the dimension reduction ratio. The vector s measures the importance of each channel of the feature map *P*. The SE block applies the scale operation to updating the input feature map *P* to a new feature map *F*, where formally, F=s⊙P. This scaling mechanism enhances FPN’s responsiveness to informative features, resulting in *SE-enhanced* feature maps, $F_{i}\;\left(3\leq i\leq 7\right)$.

#### 3.2.3. Generation of Pixel-Level Masks

Although the SE-enhanced features $\left\{F_{i}\right\}$ effectively capture multi-scale patterns, they remain sensitive to environmental noise—such as water condensation and reflections from petri dishes—commonly present during radicle imaging. These artifacts often produce false edge responses that mimic true radicle boundaries, leading to segmentation errors. To overcome this challenge, the final stage of our Pixel-level Feature Extraction module incorporates a decoder designed to explicitly model geometric primitives underlying embryonic axis development, including linear extension trends and curvature transitions. This shape-aware representation enables the network to extract intrinsic structural patterns from multi-scale features, thereby enhancing robustness against noise-induced false boundaries.

Inspired by BlendMask [48], the pixel-level decoder of GLEN is learn parameters from $\left\{F_{i}\right\}$ and output corresponding pixel-level masks. However, lower-level features have excessive spatial resolution, which increases computational complexity, while higher-level features lack the spatial precision needed for fine boundary delineation. To balance computational efficiency with feature richness, we feed only $\left\{F_{3},F_{4},F_{5}\right\}$ into this decoder, which outputs a pixel mask *B* for each *k*-th channel across all the three input feature maps of $\left\{F_{3},F_{4},F_{5}\right\}$. The process of the decoder to generate the masks *B* is formulated into Equations (4)–(6) along with the dimension evolution.

(4) $$\begin{array}{ccc} {\tilde{F}}_{i} & =\mathrm{ReLU}(\mathrm{BN}\left({\mathrm{conv}}_{3\times 3}\left(F_{i}\right)\right))\;,\;i=3,4,5 & {\tilde{F}}_{i}\in R^{256\times \frac{H}{2^{i}}\times \frac{W}{2^{i}}} \end{array}$$

(5) $$\begin{array}{ccc} x & ={\tilde{F}}_{3}+\mathrm{up}\left({\tilde{F}}_{4}\right)+\mathrm{up}\left({\tilde{F}}_{5}\right) & \;x\in R^{256\times \frac{H}{8}\times \frac{W}{8}} \end{array}$$

(6) $$\begin{array}{ccc} B & ={\mathrm{conv}}_{1\times 1}\left({\mathrm{conv}}_{3\times 3}\left(\mathrm{up}\left({\mathrm{conv}}_{3\times 3}^{n}\left(x\right)\right)\right)\right) & B\in R^{k\times \frac{H}{4}\times \frac{W}{4}} \end{array}$$

In a batching and nonlinear way, Equation (4) normalizes the channel dimensions of the three SE-enhanced feature maps to 256. In the process defined by Equation (5), $F_{4}$ and $F_{5}$ are first upsampled to match the spatial resolution of $F_{3}$, which is of size $\frac{H}{8}\times \frac{W}{8}$ at each channel. The output x can then be obtained by element-wise summation of $F_{3}$ and the upsampled $F_{4}$ and $F_{5}$. Equation (6) defines the process of generating *B* (i.e., *k*-channel pixel mask). Specifically, a sequence of *n* 3×3 convolutions is applied on x to fuse multi-scale information. The resulting feature map is upsampled with a factor of two, and subsequently passes through a 3×3 convolution followed by a 1×1 convolution, which together outputs $B\in R^{k\times \frac{H}{4}\times \frac{W}{4}}$. In Equation (6), *n* and *k* are two hyperparameters and they are set to 3 and 4, respectively, in the implementation of GLEN. Essentially, the *k* masks emphasize salient morphological features.

The right part of Figure 7 visualizes the features maps generated at different stages of GLEN’s Pixel-level Feature Extractor, using an input image photographing five elm seedlings as an example. In particular, it presents a zoomed-in view of a single-channel mask, where five radicles as well as their curved structures are distinctly highlighted by our Pixel-level Feature Extractor, demonstrating clear correspondence with those in the original input image. In summary, the design of our Pixel-level Feature Extractor paves the way for accurate and detailed representation of fine-grained morphological traits, such as radicle region and curvature.

### 3.3. Instance-Level Feature Extraction

**Figure 8:**
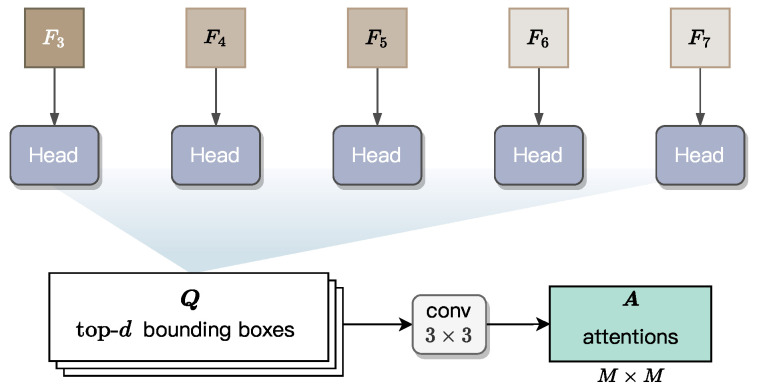
Structure of the Instance-level Feature Extractor in GLEN.

The feature maps $\left\{C_{i}\right\}$, $\left\{P_{i}\right\}$ and $\left\{F_{i}\right\}$ all represent pixel-level information, which limits their ability to capture radicles as individual instance or object. However, accurate length measurement requires the instance-level, spatial information of each individual radicle. To this end, we integrate into GLEN an Instance-level Feature Extractor, whose structure is illustrated in Figure 8. This module first predicts a bounding box for each radicle based on $\left\{F_{i}\right\}$, and then derives attention maps within the predicted bounding boxes. These maps encode instance-level information, including instance shape and spatial position, thereby enabling more precise length estimation. The design of this module follows the architecture of BlendMask, adapted here to suit our fine-grained and accurate length measurement.

#### 3.3.1. Prediction of Bounding Boxes

For each pixel-level feature map $\left\{F_{i}\right\}$ with 3≤i≤7, a head block is applied to predict a set of bounding boxes. All the five heads are learnable and share the same structure. This head block is adapted from the head design in the FCOS model [51], a single-stage object detector known for its efficiency and accuracy. As the original FCOS head generates dense bounding box predictions across all spatial locations, we retain only the top-*d* high-confidence box predictions to reduce computational overhead. These boxes are denoted as $\left\{Q_{j}\right\}$ with 1≤j≤d. Subsequently, the Non-Maximum Suppression (NMS) approach is applied to these *d* bounding boxes, leaving a box for each radicle instance.

Specifically, each bounding box is defined by a five-tuple, (π;l,t,r,b), to localize the instance boundary, where π is a location within the box, and the remaining four values—*l*, *t*, *r*, and *b*—represent the distances from π to the left, top, right, and bottom edges of the bounding box, respectively. Obviously, once these values are regressed by the head block, they uniquely define a bounding box for a radicle instance.

#### 3.3.2. Generation of Instance Attentions

As illustrated in Figure 8, we apply a 3×3 convolutional block with M×M output channels to the top-*d* predicted bounding boxes, generating an attention map of spatial resolution M×M for each bounding box. In the implementation of GLEN, *M* is a fixed hyperparameter set to 7, defining the spatial resolution of instance attention maps. The operation flow in Figure 8 is used during GLEN inference. In contrast, during training, ground-truth bounding boxes are directly used to generate the corresponding attention maps for supervision.

These attention maps act as spatial priors, emphasizing regions of interest (RoIs) within each bounding box that are most indicative of radicle morphology. The weighting mechanism in these attention maps operates directly on the instance-level features obtained after RoI alignment, measuring how high-activation areas consistently align with the true radicle axis while effectively suppressing irrelevant background features and adjacent non-radicle tissues. By integrating these maps into the instance-level feature refinement process, GLEN enhances its ability to isolate and preserve morphological cues that are critical for accurate length estimation in real-world scenarios, where visual noise or curvature may obscure radicle boundaries.

### 3.4. Radicle Length Prediction

**Figure 9:**
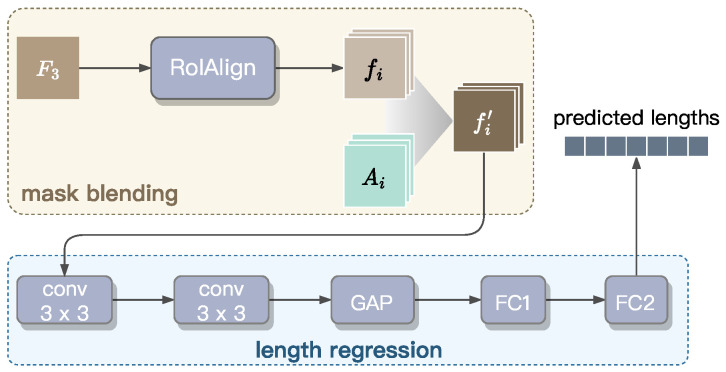
Structure of the Radicle Length Predictor of GLEN.

This section establishes the Radicle Length Predictor designed to perform instance segmentation masks and radicle length measurement. As shown in Figure 9, this predictor consists of two integrated components: (1) a mask blending module for precise boundary delineation, and (2) a regression module for accurate length estimation. To improve computational efficiency, we extract pixel-level features only from the $F_{3}$ feature map, because it retains highest spatial resolution and offers richer pixel-level information compared to the other levels of the feature pyramid, as illustrated in Figure 7.

**Mask blending**. The blender module is responsible for instance mask prediction. First, $F_{3}$ is fed into a RoIAlign block to extract the basis features $f_{i}$ for $1\leq i\leq n_{\mathrm{NMS}}$, which represent RoIs and encode position-sensitive semantic information. Here, $n_{\mathrm{NMS}}$ denotes the number of bounding boxes retained after applying NMS to the top-*d* highest-scoring boxes. These basis features $f_{i}$ are then blended with attention maps $A_{i}$, generated by the Instance-level Feature Extractor to capture high-level morphological characteristics of individual radicles. This blending results in a new mask $f_{i}^{\prime }=f_{i}⊙A_{i}$, where ⊙ denotes element-wise multiplication. The enhanced mask $f_{i}^{\prime }$, with a precise boundary for each radicle instance, are then passed through the regression module for length prediction.

**Length regression**. For length regression, we propose a convolutional network composed of five blocks. Initially, the input masks $f_{i}^{\prime }$ are processed through two 3×3 convolutional layers, each followed by batch normalization and ReLU activation, to refine localized features. Subsequently, global average pooling (GAP) is used to condense and flatten the spatial features into an $n_{\mathrm{bat}}\times 64$ matrix, where $n_{\mathrm{bat}}$ is the number of radicle instances within a batch. This matrix is then passed through two fully connected layers (i.e., FC1 and FC2) to produce the final regression output, $\hat{l}$, a 64-dimension vector in which the *i*-th value represents the predicted length of the radicle corresponding to the basis feature $f_{i}$. The dimension of $\hat{l}$ is set to 64, as a petri dish typically contains fewer than 64 seedlings. The value of ${\hat{l}}_{j}$ is set to zero if *j* exceeds the number of seedlings present within the input image.

### 3.5. Model Training

The proposed GLEN adopts a two-stage progressive training strategy to jointly optimize radicle feature extraction and length prediction. This sequential approach is necessitated by the architectural dependency between tasks: the length prediction module operates exclusively on boxed radicle features produced by the two feature extractors. Simultaneous training could lead to task interference, as regression gradients might disrupt the learning of spatially sensitive features in the backbone before they have adequately stabilized. Real-time data augmentation and preprocessing routines were applied throughout the training process to enhance data diversity and model compatibility. In Stage 1 of training, the objective is to minimize the combined loss of the Pixel-level Feature Extractor and the Instance-level Feature Extractor. This loss, denoted as $L_{S1}$, is calculated by

(7) $$L_{S1}=\lambda_{1}\cdot L_{\mathrm{pixel}}+\lambda_{2}\cdot L_{\mathrm{instance}}$$

To compute the pixel-level loss $L_{\mathrm{pixel}}$, we incorporate two components: mask loss and semantic loss. The mask loss is calculated using binary cross-entropy (BCE) between each feature map $F_{i}$ (for 3≤i≤7) and the ground-truth mask $y_{m}$, penalizing pixel-wise classification errors. The semantic loss employs cross-entropy (CE) to evaluate ${\mathrm{model}}^{\prime }s$ performance in classifying each pixel into its corresponding semantic category, where $y_{c}$ denotes the ground-truth class labels and $p_{c}$ represents the predicted probabilities. Together, these components guide model to accurately distinguish object boundaries and class identities at the pixel level. The instance-level loss $L_{\mathrm{instance}}$ consists of three key components: object existence confidence evaluated via CE $\gamma (p_{o})$, localization accuracy measured by $\left(1-\mathrm{IoU}\left(Q,\hat{Q}\right)\right)$, and instance segmentation quality quantified through BCE $\left(s_{\pi },{\hat{s}}_{\pi }\right)$.

To further improve the accuracy of radicle mask prediction in object detection, we introduce an instance-level detection loss into $L_{S1}$. This loss is composed of three components: focal loss, box loss, and centerness loss. These are computed using cross-entropy, intersection-over-union (IoU), and binary cross-entropy, respectively. The focal loss, a widely used variant of cross-entropy for object detection tasks, is defined as $-\frac{1}{n_{\mathrm{pos}}}\sum_{\left(x,y\right)}{\left(1-p_{o}\right)}^{\gamma }\log\left(p_{o}\right)$, where $n_{\mathrm{pos}}$ denotes the number of positive samples, $p_{o}$ is the predicted probability of an object being present at pixel location (x,y), and γ, set to two in our implementation, is the focusing parameter. This focal loss aims to handle class imbalance by down weighting to make training focus on negatives. The box loss uses an IoU-based metric to measure the spatial discrepancy between the predicted bounding box $\hat{Q}$ and the ground truth Q. Obviously, a perfect overlap results in zero loss. The centerness loss evaluates how close a predicted pixel lies to the center of a ground-truth object. Here, $s_{\pi }$ denotes the ground-truth centerness score at a pixel, which quantifies the proximity of the pixel to the object’s center. This term helps suppress low-quality predictions with bounding boxes that deviate significantly from the object center.

The Radicle Length Predictor is trained in Stage 2, where the two well-trained feature extractors are frozen. The loss function is defined as

(8) $$L_{S2}=\frac{1}{n_{\mathrm{bat}}}\sum_{i=1}^{n_{\mathrm{bat}}}\left\{\begin{array}{cc} {\left(\ell_{i}-\hat{\ell_{i}}\right)}^{2}/2\beta , & |\ell_{i}-\hat{\ell_{i}}|<\beta \\ |\ell_{i}-\hat{\ell_{i}}|-\beta /2, & \mathrm{otherwise}\;, \end{array}\right.$$

where $n_{\mathrm{bat}}$ is the batch size and β is a threshold parameter. In the design of loss function $L_{S2}$, we adopt a hybrid supervision policy to compare the predicted radicle lengths ${\hat{\ell }}_{i}$ with the ground-truth lengths $\ell_{i}$ for the *i*-th sample in a batch. When the prediction error is small (i.e., the residual is below β), the loss behaves quadratically, similar to L2 loss, thereby encouraging precise fitting. For larger residuals, the loss transitions to linear penalization, similar to L1 loss, which mitigates the influence of outliers and prevents excessive sensitivity. This L1-like component also contributes to smoother gradients, particularly beneficial during the early stages of training. During calculating $L_{S2}$, the transition between L2-like and L1-like behaviors is controlled by the threshold β, which is empirically set to 1.0 in our GLEN implementation to strike a balance sensitivity and robustness.

## 4. Evaluation

### 4.1. Experimental Setup

We implemented GLEN using Python 3.8.10 and trained it on a computer equipped with an Intel(R) Xeon(R) CPU @ 2.30 GHz, 251 GB RAM, and an NVIDIA RTX 2060 SUPER GPU with 8 GB VRAM. The training environment was based on PyTorch 1.10.0, with CUDA 11.3 and cuDNN 8.2.0. The hyperparameters for training GLEN are outlined in Table 2.

### 4.2. Performance Metrics

#### 4.2.1. Evaluation Metrics

To systematically evaluate the performance of the GLEN model, we assess its two core tasks: radicle instance segmentation and length prediction. Segmentation performance is measured using metrics that capture localization accuracy and boundary consistency, while length prediction is evaluated based on the degree of agreement between predicted and ground-truth measurements.

For segmentation performance, we report **mAP** (mean Average Precision), **AP50** (Average Precision at 50% IoU), and **AP75** (Average Precision at 75% IoU). For length prediction accuracy, we report **MAE** (mean absolute error), **RMSE** (root mean squared error), **Pearson correlation coefficient**, and **R^2^** (coefficient of determination). These selected metrics are widely adopted in computer vision and regression tasks, ensuring both scientific rigor and a comprehensive assessment of the model’s capabilities.

#### 4.2.2. Baseline Method

We compare our GLEN model with a representative class of recent approaches based on the U-Net architecture, which have been widely adopted in the past few years for plant phenotyping tasks. These methods typically use a U-Net model to segment radicles in images, followed by a skeletonization algorithm to estimate radicle length. In our evaluation, we replicate this standard pipeline: we apply U-Net to identify the radicle regions and then compute the length by counting the number of pixels along the skeleton of each segmented region. Since this pixel-counting process is deterministic and implemented via standard image processing tools, this measurement is reliable. In other words, the error in the overall pipeline predominantly originates from U-Net segmentation rather than the skeleton-based measurement itself. For convenience, we refer to this pipeline as “U-Net” throughout the evaluation.

### 4.3. Results and Analyses

#### 4.3.1. Training Convergence

The model employs a standard 80:20 train–validation partition, consistently applied across the distinct datasets used in each training stage. Training is conducted in two sequential stages. In Stage 1, with the ResNet-50 backbone initialized using pre-trained weights from the AdelaiDet project [52], the Pixel-level and Instance-level Feature Extractors are jointly optimized using the stochastic gradient descent (SGD) with momentum. In Stage 2, these two extractors are frozen, and only the length prediction module is trained, using the same optimizer configuration. During the refinement phase of length prediction, SGD is used to fine-tune exclusively the parameters of the regression procedure of radicle lengths. Momentum acceleration is maintained to enhance convergence, and gradient clipping is applied to stabilize training by preventing excessively large parameter updates.

**Figure 10:**
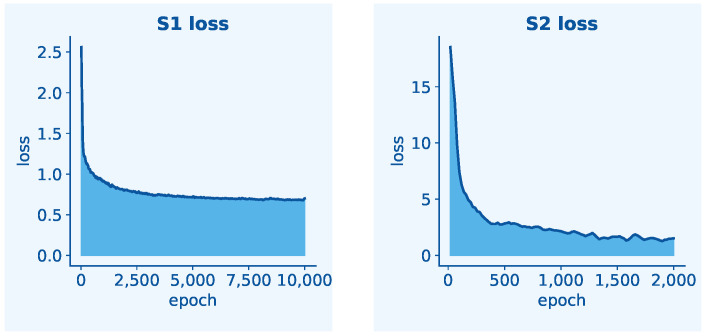
Convergence of GLEN during the two-stage training.

Figure 10 plots the convergency performance of GLEN. As shown in the left part of Figure 10, the joint loss $L_{S1}$ decreases sharply in the initial epochs, indicating the model’s rapid learning of fundamental features. The curve then converges to a stable, non-zero value of approximately 0.7. This stabilization is expected, as $L_{S1}$ is designed to jointly optimize both pixel-level and instance-level predictions. The residual loss primarily reflects the inherent complexity and high variability of the instance-level task, which poses a greater challenge than simple pixel identification. This trend demonstrates the stable convergence of our feature extractors on a complex, multi-objective task. The right part of Figure 10 illustrates the loss curve during Stage 2 training of the GLEN model, where the Radicle Length Predictor is optimized while the two feature extractors remain frozen. The loss drops sharply within the first 250 epochs—from above 18 to below 4—indicating rapid convergence and effective alignment of the regression module with the extracted features. Subsequently, the loss declines more gradually, stabilizing between epochs 500 and 2000, reflecting progressive refinement and convergence. This loss curve demonstrates that the pre-trained feature representations are sufficiently informative for the downstream regression task and that the length predictor effectively captures geometric cues essential for accurate radicle length measurement.

#### 4.3.2. Model Performance

**Figure 11:**
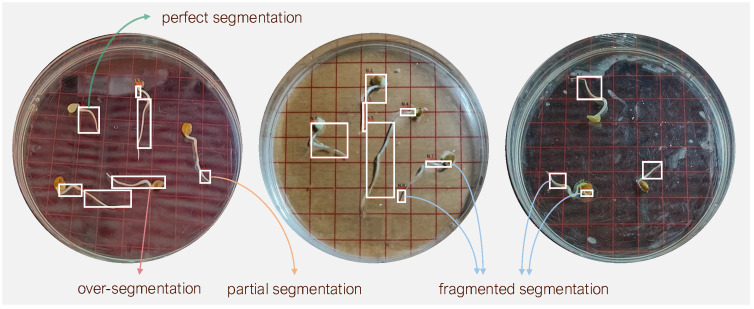
Examples of radicle segmentation produced by U-Net.

**Segmentation performance**. Table 3 quantifies marked differences across models. For bounding box detection, GLEN outperforms all baselines, achieving 83.9% AP50 and 66.4% AP, representing absolute improvements of 29.4 and 22.4 percentage points, respectively, over U-Net’s baseline scores of 54.5% (AP50) and 44.0% (AP75). For instance segmentation, GLEN with SE attains 82.5% AP50 and 52.0% AP75, surpassing U-Net by 29.3 and 6.5 percentage points, respectively. Comparison with the ablation further highlights the contribution of SE blocks, yielding improvements of 2.9% in bounding box AP50, 2.2% in bounding box AP75, and 3.9% in segmentation AP50, while maintaining comparable performance on segmentation AP75. Figure 11 illustrates that U-Net performs better on short radicles. However, it also presents representative failure cases in radicle segmentation, showing key limitations of U-Net in achieving accurate instance-level delineation. In more than half of the examples, the model fails to produce correct instance segmentation. Over-segmentation occurs when the adjacent cotyledon is erroneously included as part of the radicle. Another issue is partial segmentation, where only a portion of the radicle is captured. Additionally, fragmented segmentation is observed, wherein a single radicle is incorrectly identified as multiple distinct instances, each assigned a separate bounding box. Notably, these fragmentation errors frequently occur at points of curvature or deviation, suggesting that three-dimensional geometric traits of radicle—manifesting as discontinuities in the two-dimensional image plane—pose a significant challenge for instance segmentation.

**Length prediction accuracy.** As quantified in Table 4, GLEN achieves the best overall performance. It achieves superior regression performance, with a mean absolute error (MAE) of 3.83 mm—representing a 55% reduction compared to U-Net’s 8.44 mm—and a root mean squared error (RMSE) of 5.03 mm, reflecting a 47.5% decrease from U-Net’s 9.59 mm. The Pearson correlation coefficient improves substantially from 0.817 (U-Net) to 0.945 (GLEN), indicating strong linear agreement with ground truth. The coefficient of determination (R^2^) also increases markedly, from 0.406 to 0.793. Compared to GLEN without SE, the inclusion of SE blocks results in modest gains: a 4.3% reduction in MAE (from 4.00 mm to 3.83 mm), a 1.0% reduction in RMSE (from 5.08 mm to 5.03 mm), and a slight increase in R^2^ (from 0.788 to 0.793), while maintaining an identical Pearson correlation of 0.945. These results confirm the effectiveness of SE modules in enhancing regression accuracy and model robustness.

Based on the table data, a notable performance gap exists when removing attention mechanisms from GLEN. The variant “GLEN without attention” shows significantly degraded results compared to the full GLEN. Its MAE jumps to 6.96 mm, an increase of 82% over the full model’s 3.83 mm, while RMSE increases to 8.47 mm, reflecting a 68% increase from the full model’s 5.03 mm. Both correlation metrics decline substantially: the Pearson correlation coefficient decreases to 0.877 compared to the full GLEN’s 0.945, and the coefficient of determination (R^2^) drops markedly to 0.412, representing a 48% reduction from the full model’s 0.793. This performance deficit confirms the critical role of attention mechanisms in measurement precision and predictive consistency. The attention mechanism is indispensable for capturing spatial dependencies essential for accurate length regression.

**Figure 12:**
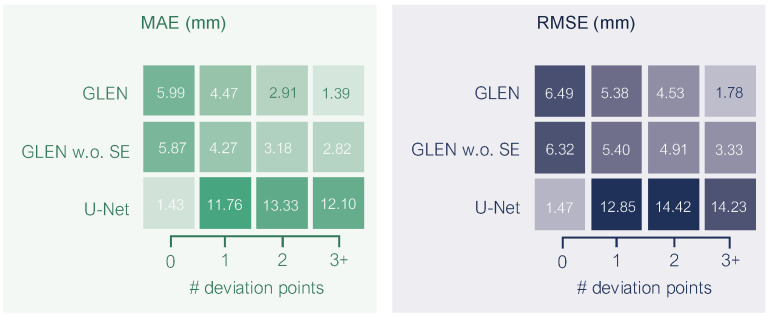
Prediction performance across seedlings grouped by number of deviation points.

**Performance variation by deviation points**. Figure 12 illustrates how prediction accuracy varies with radicle curvature, as grouped by the number of deviation points. In the 0-deviation-point group, where radicles are straight, U-Net achieves the lowest mean absolute error (MAE) of 1.43 mm and RMSE of 1.47 mm, reflecting its strength in handling two-dimensional structures (often presented on short radicles) via skeleton-based measurement, which estimates length by counting the number of pixels traversed along the radicle. However, its performance degrades substantially for curved radicles, whose proportion is beyond 90% in our GermElmData dataset. In contrast, GLEN demonstrates robust generalization across increasing morphological complexity. For seedlings with one deviation point, GLEN attains an MAE of 4.47 mm (62% lower than U-Net) and an RMSE of 5.38 mm (58% lower than U-Net). The advantage becomes more pronounced with increasing curvature: at two deviation points, it reduces MAE by 78% and at three or more, by 88.5%, respectively. These results highlight the curvature-aware learning capability of GLEN and underscore the limitations of traditional skeletonization-based methods under complex geometric variation. Comparing GLEN and its ablation, we observe that GLEN performs slightly worse in the 0-deviation-point group, suggesting that the SE blocks yield only marginal improvement for structurally simple cases. Nonetheless, the inclusion of SE blocks significantly improves performance as geometric complexity increases, due to their ability to enhance the model’s sensitivity to local contextual features.

**Figure 13:**
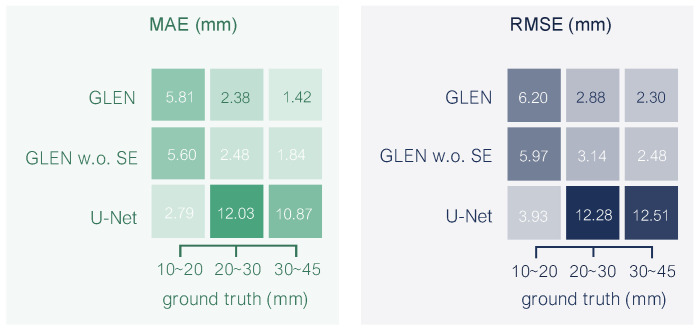
Prediction performance across seedlings grouped by ground-truth radicle lengths.

**Performance variation by radicle length**. Figure 13 presents the prediction performance of each model across seedling groups categorized by their ground-truth radicle length. In the shortest group (10∼20 mm), U-Net achieves the lowest MAE (1.13 mm) and RMSE (1.28 mm), as almost all seedlings in this range present no curvatures. In contrast, both GLEN and its ablation exhibit higher errors in this group, with GLEN slightly under-performing its ablation—consistent with the curvature-dependent pattern observed in Figure 12. As radicle length increases, GLEN and its ablation maintain stable and low error rates, while U-Net’s performance deteriorates markedly. In the longer groups, GLEN achieves the best overall accuracy, reducing MAE and RSME by 80∼87% compared to U-Net. These results underscore the robustness of GLEN in handling longer and more structurally complex seedlings, while highlighting the benefits of the SE blocks under such conditions.

## 5. Discussion

The GLEN model represents a significant advancement in automated phenotypic analysis of plant seedling morphology by effectively integrating deep learning architectures with targeted biological insights. Through its dual-path approach, combining pixel-level and instance-level feature extraction enhanced by SE blocks, GLEN successfully addresses the challenges posed by the inherent complexity of curved radicle structures. Traditional approaches, including widely adopted frameworks like U-Net, generally rely on simplified skeletonization or linear approximations, which substantially underperform when measuring radicles exhibiting significant curvature or multiple deviation points. Our experimental results underscore GLEN’s capability to deliver millimeter-scale accuracy, dramatically outperforming conventional models by nearly 60% in mean absolute error. Additionally, the introduction of synthetic data generated via AI-guided morphological guidance provided a scalable and efficient strategy to augment morphological variability, reduce reliance on extensive manual annotation, and improve model generalization capabilities. The GLEN methodology facilitates rapid, precise phenotypic assessments essential for breeding programs aimed at developing disease-resistant and stress-resilient elm cultivars. Moreover, the approach provides a foundational framework readily adaptable to other phenotyping tasks involving complex morphological features, thus holding broader significance for precision agriculture, ecological monitoring, and biological research.

The GLEN framework holds significant potential for real-world deployment in seed testing laboratories and breeding facilities. The final trained GLEN model, at 142 MB and processing a single elm germination image in approximately 1200 ms, can be integrated into dedicated imaging stations or portable devices equipped with cameras to enable high-throughput, batch processing of germinating seeds. This capability offers a robust platform for automated seed vigor assessment, providing the rapid, precise, and objective measurement of radicle length essential for this critical task.

Despite these successes, certain limitations must be acknowledged. First, GLEN was trained primarily on elm seed data captured under controlled laboratory conditions. Consequently, performance could potentially diminish in field environments characterized by variable lighting, occlusions, and diverse environmental factors, or when extended to morphologically distinct plant species. Second, while GLEN demonstrates reasonable computational efficiency, further optimization may be required for real-time deployment in resource-limited or edge computing environments commonly found in practical agricultural settings. Third, GLEN’s current design and testing might not adequately address scenarios where seedlings are densely placed, with significant overlaps or occlusions. These conditions could complicate instance segmentation, leading to decreased model performance. This limitation stems from the model’s current difficulties in disentangling overlapping structures. Mitigating this limitation necessitates strategies focused on enhancing segmentation robustness under such conditions. Promising approaches include augmenting the training data with diverse, realistically complex scenes exhibiting dense arrangements and variable overlap patterns, investigating deep learning architectures intrinsically designed to model spatial relationships between neighboring instances in cluttered environments, or employing imaging systems capable of capturing multiple perspectives to resolve ambiguities inherent in single-view images. Fourth, variations in image acquisition protocols (e.g., camera type, angle, resolution, background substrates, and lighting intensity) could affect the model’s generalization performance, necessitating standardized imaging conditions or additional domain adaptation. Fifth, a key limitation arises because GLEN predicts morphological features in pixel units. Accurately converting these predictions to real-world measurements requires a reliable scaling factor. While including a printed grid in the image provides a robust solution for establishing this scale, our current method lacks a robust, integrated approach for deriving the necessary pixel-to-physical conversion ratio when such an explicit reference object is absent. This necessitates the future use of alternative strategies, such as utilizing precise camera specifications (e.g., focal length, sensor size) combined with measured working distances, or incorporating a calibration object of known dimensions within the field of view.

Future work could focus on expanding the diversity and complexity of training datasets, by incorporating additional plant species and images captured under varied, real-world conditions. Methodological improvements targeting computational efficiency and lightweight model desgin would further broaden GLEN’s applicability, facilitating its deployment in portable, real-time phenotyping systems. Additionally, integrating temporal growth dynamics into the GLEN framework may offer deeper insights into developmental trajectories and phenotypic plasticity, advancing the understanding of plant responses to diverse environmental stressors.

## 6. Conclusions

This paper presents GLEN, a deep learning framework developed alongside the GermElmData dataset for automated measurement of curved radicles in germinating elm seeds. GLEN addresses key limitations of conventional methods, which often approximate curved structures as linear segments and fail to capture critical morphological traits. To the best of our knowledge, GLEN is the first end-to-end framework that employs machine learning techniques for fully automated measurement of seedling germination traits without human intervention. This framework demonstrates strong potential to advance precision phenotyping in both agricultural research and ecological monitoring. In the future, we will focus on improving the GLEN’s generalizability, computational efficiency, and adaptability to dynamic environmental conditions, thereby extending its applicability to a broader range of plant phenotyping scenarios.

## Figures and Tables

**Table 1:** Segmentation performance: real vs. synthetic training data.

Training Data	bbox AP50	segm AP50
Real Images	81.1%	79.4%
Synthetic Images	77.4%	74.9%

**Table 2:** Parameters for GLEN training.

Parameter	Description/Value	Parameter	Description/Value
batch size	4	input size	[640, 800] (multi-scale)
learning rate (Stage 1)	$5\times 10^{-3}$	$\lambda_{1}$ (in $L_{S1}$ loss)	1.0
learning rate (Stage 2)	$1\times 10^{-2}$	$\lambda_{2}$ (in $L_{S1}$ loss)	1.0
weight decay	$1\times 10^{-4}$	γ (in $L_{S1}$ loss)	2.0
momentum	0.9	*k* (mask channels)	4
number of iterations (Stage 1)	$1\times 10^{4}$	*M* (attention resolution)	7×7
number of iterations (Stage 2)	$2\times 10^{3}$	*r* (SE reduction ratio)	16
number of warmup iterations	$1\times 10^{3}$	gradient clipping	1.0 (max norm)
NMS threshold	0.3	β (in $L_{S2}$ loss)	1.0

**Table 3:** Comparison in segmentation performance.

Model	bbox AP50	bbox AP75	segm AP50	segm AP75
U-Net	54.5%	44.0%	53.2%	45.5%
GLEN without SE	81.0%	64.2%	78.6%	52.6%
GLEN	83.9%	66.4%	82.5%	52.0%

**Table 4:** Comparison in length prediction performance.

Model	MAE (mm)	RMSE (mm)	Pearson	R^2^
U-Net	8.44	9.59	0.817	0.406
GLEN without SE	4.00	5.08	0.945	0.788
GLEN without attention	6.96	8.47	0.877	0.412
GLEN	3.83	5.03	0.945	0.793

## Data Availability

Full dataset is available on request.
